# Surface modification of MXene using cationic CTAB surfactant for adsorptive elimination of cefazolin antibiotic from water

**DOI:** 10.1038/s41598-025-01435-y

**Published:** 2025-05-12

**Authors:** Jafar Abdi, Golshan Mazloom, Yeojoon Yoon

**Affiliations:** 1 Faculty of Chemical and Materials Engineering, Shahrood University of Technology, 3619995161 Shahrood, Iran; 2 Center for International Scientific Studies and Collaborations, Tehran, Iran; 3 Department of Chemical Engineering, Faculty of Engineering, University of Mazandaran, 47416-13534 Babolsar, Iran; 4 Department of Environmental and Energy Engineering, Yonsei University, Wonju, Republic of Korea

**Keywords:** MXene, CTAB, Surface modification, Cefazolin antibiotic, Adsorption, Wastewater treatment, Chemical engineering, Environmental chemistry

## Abstract

The increasing prevalence of pharmaceutical contaminants in aquatic ecosystems has raised significant environmental concerns, necessitating the development of efficient removal strategies. In this study, Ti_3_C_2_T_x_ MXene was synthesized and modified with cetyltrimethylammonium bromide (CTAB) to enhance its adsorption performance for cefazolin (CFZ), a widely used cephalosporin antibiotic. The structural and physicochemical properties of the modified MXene were comprehensively characterized using FESEM, EDS, FTIR, XRD, BET, and zeta potential analyses. Adsorption experiments were conducted under various operational conditions, including pH, contact time, adsorbent dosage, and initial CFZ concentration. The results revealed that CTAB modification significantly improved the adsorption capacity by increasing interlayer spacing and enhancing the accessibility of active adsorption sites. The optimized adsorbent (MC-0.9) exhibited a maximum CFZ removal efficiency of 96.3% and an adsorption capacity of 481.5 mg/g under optimal conditions: an adsorbent dosage of 0.1 g/L, a solution pH of 5, a contact time of 60 min, and an initial CFZ concentration of 50 mg/L. Kinetic and isotherm modeling indicated that the batch adsorption process followed the pseudo-second-order kinetic model and fitted well with the Langmuir isotherm, suggesting monolayer adsorption. Additionally, the presence of co-existing anions adversely affected adsorption efficiency, following the order CO_3_^2−^ > Cl^−^ > SO_4_^2^^−^ > NO_3_^−^. The adsorption mechanism was primarily governed by electrostatic interactions, π–cation interactions, and hydrogen bonding. Furthermore, the CTAB-modified MXene demonstrated robust recyclability, maintaining high efficiency over four consecutive cycles, highlighting its potential as a promising adsorbent for the removal of pharmaceutical pollutants from wastewater.

**Supplementary Information:**

The online version contains supplementary material available at 10.1038/s41598-025-01435-y.

## Introduction

The use of pharmaceuticals has significantly increased with the growth of the population, leading to important environmental issues, even though their concentrations in nature are usually low (ng–µg)^1^. Their persistence and potentially harmful effects on aquatic ecosystems, human health, and wildlife have resulted in their classification as emerging pollutants^2,3^. Antibiotics are among the most used pharmaceutical categories. Cefazolin (CFZ), classified as a first-generation cephalosporin antibiotic, plays a crucial role in both human and veterinary medical practice. Its widespread application in surgical prophylaxis is attributed to its cost-effectiveness^4^. However, due to the short period of half-life, the persistence of CFZ residues in the environment remains a concern. These residues have the potential to exert adverse effects on aquatic ecosystems, including the induction of bacterial mutations and the formation of potentially harmful disinfection byproducts during wastewater chlorination processes^5^. Various remediation techniques, including electrocoagulation^6^, advanced oxidative processes (AOPs)^7–9^, and adsorption^10,11^ have been explored for eliminating CFZ from water. Each approach has its advantages and disadvantages. For instance, Esfandyari et al.^6^ noted that electrocoagulation for treating real wastewater requires a considerable amount of energy. Although AOPs are extensively studied for their effectiveness in degrading CFZ, they can be quite expensive, consume a lot of energy, and may generate unwanted by-products. On the other hand, adsorption is recognized as a straightforward, affordable, and efficient treatment option^12,13^.

Recently, two-dimensional (2D) transition metal carbides and nitrides, commonly referred to as MXenes, have attracted considerable interest in the scientific community^14,15^. The unique lamellar structure, wide lateral dimensions, and abundant functional groups enhance their hydrophilicity, large surface area, biocompatibility, active surface sites, and ability to be modified^16^. These features make MXenes particularly well-suited for use as adsorbents^17^. Additionally, MXenes can act as flexible platforms for combining metal-based and carbon-based materials, which can enhance adsorption efficiency even further^16^. Gupta et al.^18^ utilized delaminated Ti_3_C_2_T_x_ MXene to adsorb various fluoroquinolone antibiotics. The delamination process took place in coaxial cylinders within a Couette reactor, resulting in thinner and larger nanosheets. The delaminated Ti_3_C_2_T_x_ MXene demonstrated an impressive adsorption capacity, with 349 mg/g for ciprofloxacin and 290.51 mg/g for levofloxacin, attributed to its enhanced surface area. According to the represented work by Zeng et al.^19^, Ti_3_C_2_ MXene demonstrated a high surface area and contained functional groups that allow it to effectively adsorb sulfachloropyridazine antibiotics with 22.62 mg/g. This process was facilitated by chemical interactions that were influenced by surface defects and the presence of functional groups. Wang et al.^20^ synthesized alkalized MXene/ZIF composites that demonstrated remarkable adsorption capacities: 539.7 mg/g for Congo Red, 1053.3 mg/g for Tetracycline, and 7111.3 mg/g for Malachite Green. This performance could be attributed to their intercalated structure and the synergistic mechanisms involved in adsorption. Additionally, these composites were stable, recyclable, and resistant to ion interference, which enhanced their effectiveness in removing dyes and antibiotics. Liet al.^21^ developed a chitosan-based hydrogel modified with polyethyleneimine and Ti_3_C_2_ MXene, achieving impressive removal rates of chlortetracycline (91.1%) and ibuprofen (99.25%) through electrostatic interactions and hydrogen bonding. The hydrogel retained about 70% efficiency after five reuse cycles, highlighting its strong potential for wastewater treatment.

Nevertheless, MXene layers often experience restacking due to van der Waals forces, which restricts the full exposure of their active surface sites^22^. The limited spacing between the layers also restricts their ability to adsorb. To overcome these challenges, improving the adsorption efficiency of MXenes by adding interlayer spacers has proven to be a promising approach^23,24^. Recently, cetyltrimethylammonium bromide (CTAB), a type of surfactant ion, was successfully intercalated into MXenes to improve their properties. By functionalizing MXenes with CTAB, the restacking of nanosheets can be effectively prevented. Moreover, the naturally negative surface charge of MXenes over a wide pH range can negatively impact the adsorption of CFZ, since this antibiotic mainly exists as an anionic species in aqueous solutions^25^. The addition of CTAB, as a cationic surfactant, to MXene structures enhances their ability to capture anionic contaminants, improves ion exchange properties, and increases electrostatic interactions. The selection of CTAB for surface modification can be attributed to several factors, including the facile nature of the surface modification technique, enhanced adsorption selectivity, development of a homogeneous positive surface charge, and enhancement of specific surface area coupled with pore formation within the adsorbent framework^26–28^.

Despite the promising adsorption capabilities of MXenes, their practical application in removing anionic pollutants like CFZ remains underexplored. One critical challenge is their natural low interlayer spacing, which significantly reduces the accessibility of active sites for adsorption. Additionally, the negative surface charge of MXenes limits their effectiveness in adsorbing anionic contaminants such as CFZ. The performance of MXene-based adsorbents against anionic pharmaceuticals is still an underdeveloped area of research. To address these limitations, this study investigates the functionalization of Ti_3_C_2_T_x_ MXene with CTAB to enhance electrostatic interactions, prevent restacking, and improve CFZ adsorption efficiency. This novel approach provides a potential strategy for optimizing MXene-based adsorbents in water treatment applications. In this study, Ti_3_C_2_T_x_ MXene nanosheets were modified with CTAB to create a new type of MXene adsorbent. We conducted a comprehensive analysis of the physicochemical properties of the modified MXene and assessed its adsorption efficiency for CFZ under various conditions, such as contact time, pH levels, adsorbent dosage, initial antibiotic concentration, and co-existence of different inorganic anions. Additionally, we applied adsorption of isotherm and kinetic models to better understand the CFZ removal process. A potential mechanism for CFZ adsorption on the modified CTAB/MXene surface was also suggested.

## Experiments

### Materials and reagents

The MAX phase (Ti_3_AlC_2_) was procured from Redox Kala Co. Hexadecyltrimethylammonium bromide (CTAB, C_19_H_42_BrN) as a cationic surfactant was supplied by Sigma-Aldrich. Hydrochloric acid (HCl, 37 wt%), hydrofluoric acid (HF, 40 wt%), methanol (CH_3_OH), and sodium hydroxide (NaOH) were obtained from the Merck company. Cefazoline was purchased from Darou Pharmaceutical Co. All chemicals were used as received, without further purification.

### Preparation of CTAB@MXene adsorbent

The CTAB@MXene adsorbent was synthesized in two steps. At first, Ti_3_C_2_T_x_ MXenes was conducted via an etching methodology wherein an aluminum layer was selectively extracted from the Ti_3_AlC_2_ MAX phase^14^. Briefly, 1 g of MAX powder was incrementally introduced into a 30 mL of HF acid solution followed by vigorous agitation (800 rpm) for 24 h at room temperature. The resultant suspension was centrifuged and rinsed several times with deionized water until a neutral pH was attained. Finally, the obtained Ti_3_C_2_T_x_ MXene powder was dried in an oven at 60 °C for 24 h.

**Fig. 1:**
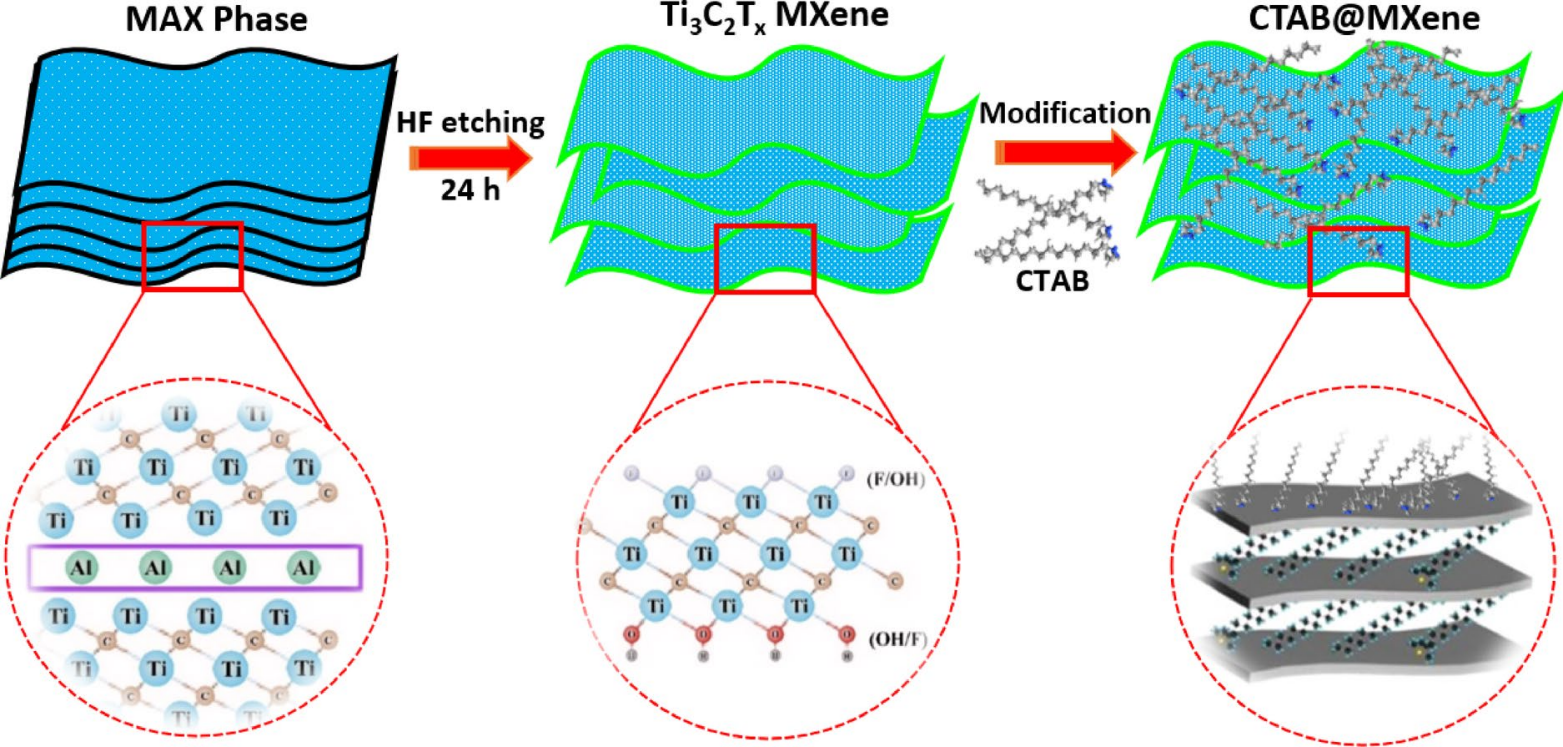
Schematic illustration of the surface modification of MXene using cationic CTAB surfactant.

In the second step, 0.2 g of the prepared Ti_3_C_2_T_x_ powder was sonicated in 20 mL of deionized water for 1 h to facilitate the exfoliation of the MXene layers. Subsequently, different amounts (2.1, 4.4, 6.5, 8.7, and 15.3 mg) of the cationic surfactant CTAB were added to the MXene suspension under continuous stirring for 20 min at room temperature. The prepared samples were designated MC-X (where X = 0.3, 0.6, 0.9, 1.2, and 2.1 mM, represents varying surfactant concentrations). Ultimately, the surface-modified Ti_3_C_2_T_x_ nanosheets were separated from the solution by centrifugation, washed three times with distilled water to remove excess and unbound surfactant, and dried in an oven at 60 °C for 12 h. The synthesis procedure of CTAB@MXene adsorbent is shown in Fig. 1.

### Characterization techniques

The synthesized samples underwent comprehensive characterization using a variety of techniques. Morphological analysis was performed using field emission-scanning electron microscopy (FE-SEM) with a Sigma 300-HV (ZEISS Co., Germany) instrument. Elemental composition was determined via energy-dispersive X-ray spectroscopy (EDS) using an EDS detector and mapping system (Oxford Instruments, UK). Fourier transform infrared (FT-IR) spectra were acquired using a PerkinElmer Spectrum One spectrometer (USA) in transmission mode, with a resolution of 4 cm^− 1^, over a range of 4000 to 400 cm^− 1^. X-ray diffraction (XRD) patterns were obtained using a PANalytical X’Pert PRO diffractometer (Germany) with Cu/Kα radiation. Specific surface area and porosity were determined using the Brunauer-Emmett-Teller (BET) method based on nitrogen adsorption/desorption isotherms at 77 K (BELSORP-mini II, BEL, Osaka, Japan). Optical properties were investigated using a UV-vis spectrophotometer (DR6000, HACH, Germany). Finally, a Litesizer zeta potential analyzer (Anton Paar) was utilized to measure the zeta potential of the adsorbents.

### Batch experiments

Model wastewater solutions containing varying CFZ concentrations (50–80 mg/L) were prepared by dissolving different quantities of CFZ in deionized water. The synthesized MXene and CTAB-modified MXene (MC-X) materials were employed as adsorbents. In each experiment, a precisely measured quantity of adsorbent (2–10 mg) was introduced into 50 mL of the CFZ solution with a specific value of pH (3–10), and the resulting mixture was agitated until equilibrium was reached. Following the separation of the solid phase, the residual CFZ concentration was quantified using a UV-Vis spectrophotometer. The performance of adsorption was assessed as a function of several parameters, including adsorbent type, adsorbent dosage, initial CFZ concentration, contact time, and solution pH. The following equations were used to determine the removal efficiency (R%) and the adsorption capacity of the MC-X at equilibrium state (q_e_):

1 $$\:R\left(\%\right)=\frac{{C}_{i}-{C}_{e}}{{C}_{i}}\times\:100$$

2 $$\:{q}_{e}=\frac{\left({C}_{i}-{C}_{e}\right)\times\:V}{m}$$

where *C*_*e*_ and *C*_*i*_ are the concentration of the remaining CFZ solution at the equilibrium and beginning of the process, *m* and *V* belong to the adsorbent mass and the volume of the solution, respectively.

## Results and discussion

### Characterization of the adsorbents

**Fig. 2:**
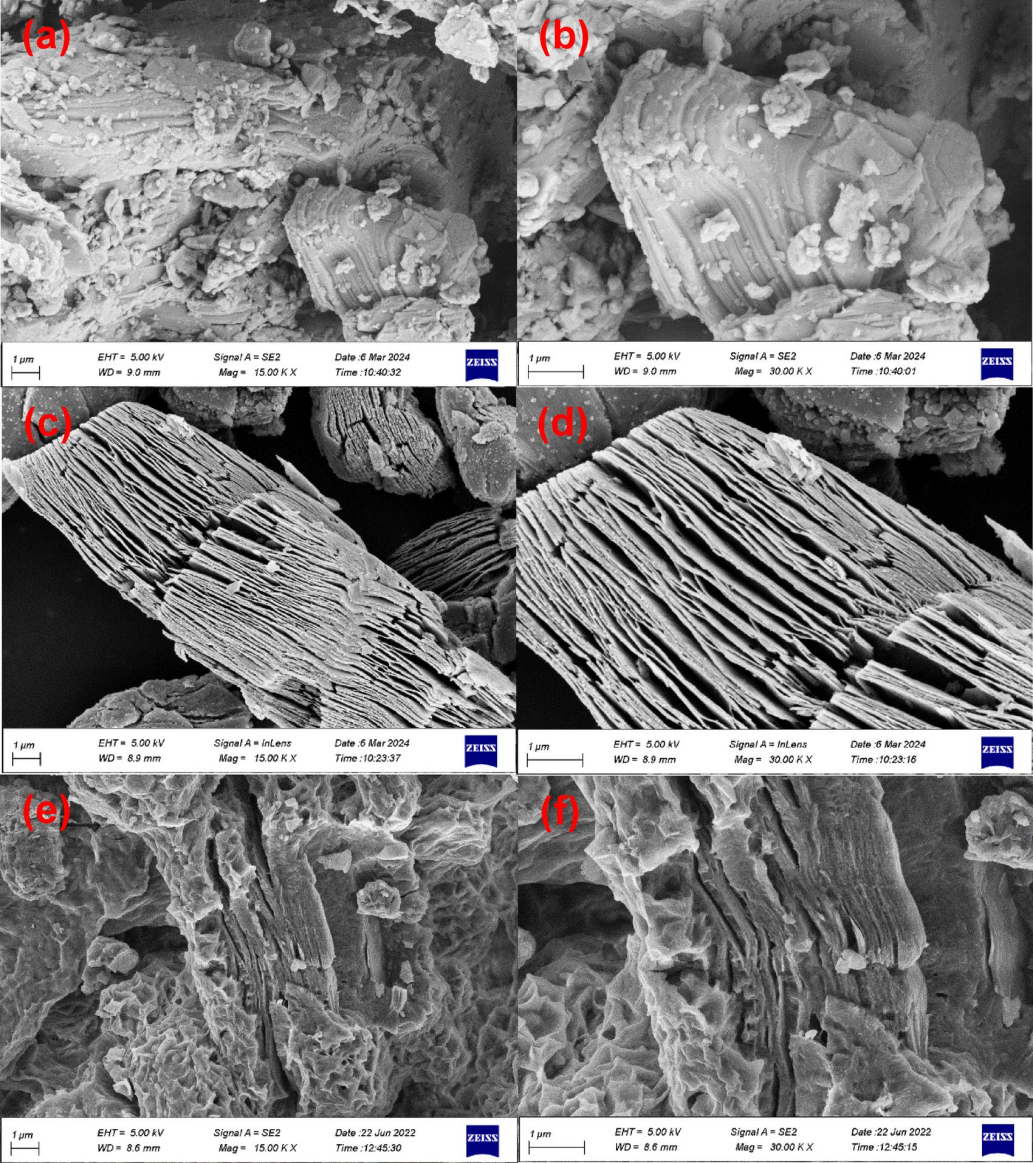
SEM images of the prepared materials in different magnifications: (**a**,**b**) MAX phase, (**c**,**d**) MXene, (**e**,**f**) CTAB@MXene.

The surface morphology and particle size of the MAX phase, MXene, and MXene modified with the cationic surfactant (CTAB@MXene) were investigated using FESEM analysis, and the images were shown in Fig. 2 in different magnifications. The images in Fig. 2a and b demonstrate that the Ti_3_AlC_2_ MAX phase exhibits a layered structure, appearing as two-dimensional sheets stacked upon each other. after 24 h of corrosion in the HF solution, the aluminum layer was etched from the MAX phase structure and open layered MXene sheets are formed. Notably, the constituent layers presented a thickness range of 20 to 60 nm, with a mean thickness of 45.3 ± 7.2 nm. The produced MXene displayed a multilayer structure characterized by a diminished stacking gap. It is hypothesized that the overall shape of the MXene, in contrast to the uniform thickness of a single layer, is the primary determinant of antibiotic adsorption efficiency. As can be seen in Fig. 2c and d, the MXene layers are separated, resembling the pages of a loosely held book. Figures 2e and f confirm that after surface modification of the MXene, the cationic surfactant CTAB covers not only the surfaces of the MXene sheets but also the spaces between the layers. This results in a change in the surface morphology of the MXene sheets, making them rougher. This increased roughness, despite a relative decrease in surface area, maintains the effectiveness and utility of the MXene for the adsorption of pollutants. EDS analysis and elemental mapping of the CTAB@MXene adsorbent were performed, and the results are presented in Fig. S1. As shown, the main components of the adsorbent sample consist of Ti, C, F, O, Al, and Br elements. The presence of bromine in the EDS spectrum of the sample, along with other analyses such as FTIR, XRD, and BET, indicates the successful attachment of the cationic surfactant CTAB onto the MXene. However, the results obtained from this analysis indicate a small amount of bromine and a significant amount of fluorine in the composition of the CTAB@MXene adsorbent.

**Fig. 3:**
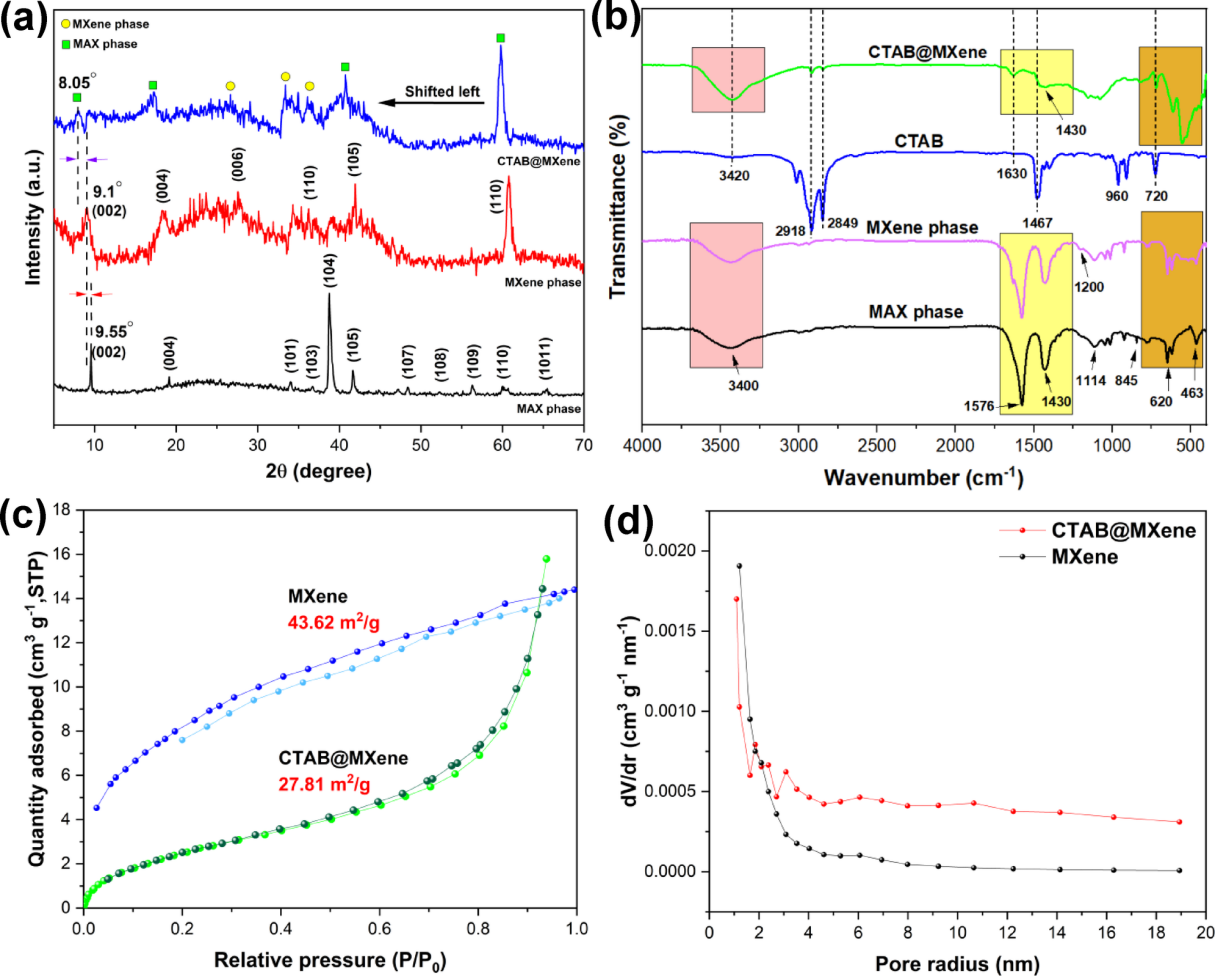
(**a**) Powder XRD patterns, (**b**) Fourier transform infrared (FT-IR) spectra, (**c**) Nitrogen adsorption-desorption isotherm at 77 K, and (**d**) zeta potential of the prepared materials.

The crystal structure of the synthesized materials was investigated using XRD analysis, and the resulting spectra were shown in Fig. 3a. The pattern of the Ti_3_AlC_2_ MAX phase shows characteristic peaks at 2θ values of 9.55°, 19.15°, 34.05°, 36.75°, 38.8°, 41.7°, 48.4°, 52.3°, 56.35°, and 60.1°, corresponding to the (002), (004), (101), (103), (104), (105), (107), (108), (109), and (110) crystallographic planes, respectively. This pattern and the diffraction of these planes confirm the synthesis of the MAX phase structure, consistent with JCPDS card No. 052–0875 ^29^. The presence of strong diffraction peaks with high intensity indicates the crystalline nature of the MAX phase structure, while the absence of additional diffraction peaks related to impurities indicates the high purity of the MAX phase. Furthermore, the formation of the Ti_3_C_2_T_x_ MXene phase was confirmed by the shift of the (002) plane peak from 2θ = 9.55° in the MAX phase structure to a lower angle of 9.1°. Using Bragg’s law, the interlayer spacing (d) in the MXene phase was calculated to be 9.71 Å, which was larger than the 9.25 Å in the MAX phase. Also, after etching aluminum from the MAX structure and forming the MXene phase, the (002) peak was broadened, indicating the separation of the layered sheets which provides a suitable space for surface modification with functional groups. After surface modification of the MXene phase with the cationic surfactant CTAB, the interlayer spacing increased from 9.71 Å to 10.96 Å, indicating the attachment of the surfactant on the surface and between the layers of the MXene sheets. It has a significant impact on pollutant adsorption due to the enhancement of active sites and more porosity. Moreover, all characteristic peaks in the MXene phase were observed in the CTAB@MXene structure, and the (002) and (004) peaks shifted. This shift was also due to the interaction of the surfactant with the surfaces and the interlayer space of the MXene sheets, which caused a change in the crystal lattice parameters^29^.

The FTIR spectra of the MAX phase, MXene, pure surfactant, and CTAB@MXene were shown in Fig. 3b. In the spectrum of the Ti_3_AlC_2_ MAX phase, absorption peaks appearing at 3400 and 1636 cm^− 1^ corresponded to hydroxyl groups in the H_2_O molecule with strong coordination bonding and external water molecules adsorbed on the surface, respectively^30^. The peaks observed at 620 and 463 cm^− 1^ were attributed to the Ti-O stretching vibration and Ti-C bond, respectively^31^. The peak at 845 cm^− 1^ corresponds to the Al-O bond. Furthermore, the spectrum of the MAX phase includes vibrational bands of C-O, C-H, and C = C at 1114, 1430, and 1576 cm^− 1^, respectively^30^. As shown in Fig. 3b, the Ti_3_C_2_T_x_ MXene has a similar vibrational pattern compared to the Ti_3_AlC_2_ MAX phase. However, minor differences are also observed. The MXene phase obtained by etching with HF did not show a peak for the Al-O bond; this indicates the successful extraction of aluminum from the MAX phase structure. Moreover, the spectrum of the MXene shows a new peak at 1200 cm^− 1^, which is attributed to the presence of the C-F functional group. The absence of this bond in the MAX phase spectrum indicated the surface coverage of the MXene sheets with the C-F functional group^32^. The presence of ammonium vibrations (3420 cm^−1^), asymmetric and symmetric stretching vibrations of N⁺-CH₃ (1630 and 1467 cm^−1^), the -CH₂ group vibrations (2918 and 2849 cm^−1^), and the out-of-plane vibration of -CH bond in the CH_3_ group (960 cm^− 1^) in the CTAB@MXene spectrum confirms the incorporation of the cationic surfactant. The positively charged quaternary ammonium groups on CTAB can promote electrostatic interactions with the negatively charged functional groups present in the cefazolin molecule (e.g., carboxylate groups). This enhanced electrostatic attraction is a crucial factor in the improved adsorption capacity of CTAB@MXene for cefazolin. The peak at 720 cm^− 1^ may be attributed to Br^−^^33^. Furthermore, the persistence of the C-F (1154 cm^−1^) and Ti-F (557 cm^−1^) bands in the CTAB@MXene spectrum indicates that the MXene basal planes retain their fluorine termination after surfactant modification^24^. These polar functional groups can also contribute to cefazolin adsorption through hydrogen bonding or dipole-dipole interactions.

The Brunauer-Emmett-Teller (BET) analysis was performed to measure the surface area and pore volume of the materials, and the results were represented in Fig. 3c and d. The obtained N_2_ adsorption/desorption isotherms for the Ti_3_C_2_T_x_ MXene and CTAB@MXene conformed to Type IV according to the IUPAC classification. The type of hysteresis indicated that the porosity had a layered structure, which was previously shown by FESEM analysis. The specific surface area for the MXene and modified MXene particles was measured to be 43.62 and 27.81 m²/g, respectively, and the pore volumes of these samples were measured to be 1.493 and 0.918 cm³/g, respectively. The decrease in surface area and pore volume after CTAB modification is attributed to the occupation of the MXene interlayer spaces and external surface by the bulky CTAB molecules. This is further supported by the shift in the pore size distribution from a narrower distribution around 3.1 nm in pristine MXene to a broader distribution centered around 10.67 nm in CTAB@MXene. This increase in average pore size, while seemingly counterintuitive for adsorption, suggests that the CTAB molecules create a more accessible environment for the relatively large CFZ molecules to interact with the functionalized surface. The larger pores in the modified MXene reduce steric hindrance, facilitating diffusion of cefazolin molecules to the active binding sites introduced by CTAB. Since CTAB occupies the MXene pore space, the specific surface area of the CTAB-modified MXene has decreased. However, the introduction of positively charged quaternary ammonium groups and hydrophobic alkyl chains of CTAB creates a greater affinity for CFZ through electrostatic interactions and potential hydrophobic interactions. These newly introduced active sites, even on a slightly reduced surface area, appear to be more effective in capturing CFZ compared to the pristine MXene surface^34^.

### Adsorption study of CFZ

#### Determination of optimum CTAB for surface modification

**Fig. 4:**
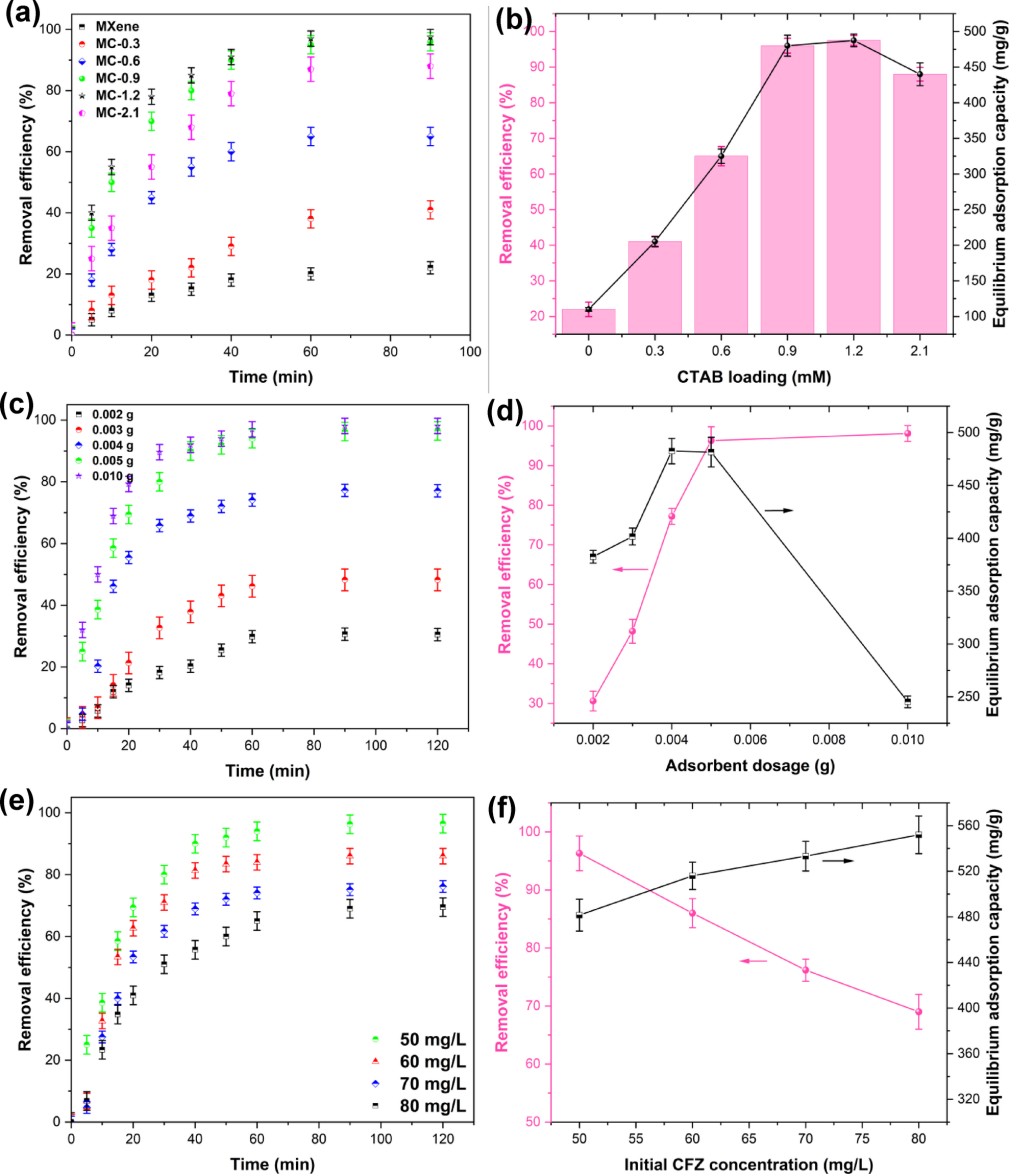
(**a**,**b**) Surface modification of MXene with various concentrations of the cationic surfactant CTAB, (**c**,**d**) effect of adsorbent dosage, and (**e**,**f**) effect of initial CFZ concentration (Experimental conditions: [CFZ]_0_ = 50 mg/L, adsorbent dosage = 5 mg, pH = 5, and V = 50 mL).

To enhance the adsorption efficiency of the CFZ antibiotic pollutant, the MXene phase was modified with various concentrations of the cationic surfactant CTAB. For the optimization of experimental parameters, a One-Factor-At-A-Time (OFAT) approach was employed. This method allowed us to systematically evaluate the impact of each variable on CFZ removal efficiency by altering one parameter while keeping others constant. In the initial stages, we also utilized a trial-and-error approach to establish a preliminary understanding of the system’s behavior and to refine the experimental ranges. The optimal concentration of CTAB was determined by adding 5 mg of the various adsorbents (MC-X samples) to 50 mL of antibiotic solution with an initial concentration of 50 mg/L at the natural pH of the solution (~ 5). As shown in Fig. 4a and b, the unmodified MXene phase could remove 22% of the CFZ pollutant. However, after surface modification with the cationic surfactant, the removal efficiency increased from 41% for MC-0.3 to 97.5% for MC-1.2. Subsequently, with a further increase in the CTAB concentration to 2.0 mM, the removal efficiency of CFZ decreased to 88%. This can be attributed to the adsorption mechanism of the surfactant on the MXene and the critical micelle concentration (CMC).

Generally, depending on the properties of the solid surface and the surfactant, as well as the surfactant concentration, different structures such as a monolayer, bilayer, or hemimicelle may be formed over the surfaces and interfaces. Some properties of the adsorbent could be changed because of surfactant adsorption. Initially, the surface wettability may be influenced. For instance, a nonionic surfactant can adsorb onto a nonpolar surface through van der Waals forces via the hydrophobic tail. In other words, by positioning the hydrophilic head towards the bulk solution, the solid surface becomes more hydrophilic^35^. A reversal of surface charge may transpire as a further mechanism. Adsorption of a cationic surfactant onto a negatively charged substrate proceeds via the initial binding of the hydrophilic head to the surface, neutralizing the charge. Subsequent adsorption promotes hydrophobic tail interactions, forming a bilayer micelle. In this configuration, the positively charged head groups orient outwards, toward the solution, thus imparting a positive charge to the surface. In other words, the adsorption process exhibits concentration dependence. When the concentration of the surfactant is low, it mainly adsorbs onto the surface in a flat configuration through electrostatic interaction. Increasing surfactant concentrations lead to monomer interactions on the surface, causing a slight delay in the observed adsorbed mass. At higher concentrations, surface aggregation occurs, with the formation of bilayers, hemimicelles, and structures resembling micelles^36^.

Given that the reported CMC value for the CTAB surfactant is 1 mM^36^, the MC-0.9 adsorbent with a removal efficiency of 96.3% performed well. It can be stated that without the aggregation of surfactant monomers on the MXene sheets, maximum utilization of the surfactant for pollutant adsorption was achieved. Increasing the surfactant concentration from 0.9 to 1.2 mM did not significantly improve the CFZ removal efficiency and only increased the by 1.2%. Therefore, 0.9 mM was selected as the optimal concentration of the cationic surfactant CTAB for surface modification of the MXene phase and the MC-0.9 sample was employed for conducting further experiments. In other words, it can be claimed that for concentrations lower than 0.9 mM, the surfactant penetrates the MXene layers, and for higher concentrations, the excess surfactant resides on the MXene surface. This can block the interlayer space, causing aggregation of surfactant monomers on the MXene sheets, forming micelles, and ultimately reducing the efficiency of the surfactant molecules and decreasing the pollutant adsorption capacity.

#### Effect of adsorbent dosage

Various amounts of optimal adsorbent were used in a batch system to investigate the effect of adsorbent dosage and contact time between the adsorbent and the pollutant solution. Figure 4c exhibits the performance of the MC-0.9 adsorbent in removing CFZ from the start of the adsorption process up to 120 min. As shown in this figure, the adsorption efficiency increased from the beginning of the process up to 60 min for all adsorbent dosages. Then no significant change was observed until the end of the adsorption time at 120 min. The increased adsorption rate observed at the beginning of the process can be attributed to the large number of unoccupied active sites on the adsorbent surface, providing ample opportunities for adsorbate binding^37,38^. Subsequently, as time progresses, the adsorption efficiency reached a constant value due to the adsorption of all CFZ molecules or the saturation of pores and active sites on the adsorbent surface. As can be seen in Fig. 4d, the adsorption efficiency increased with increasing adsorbent dosage from 2 mg to 5 mg (30.6–96.3%), and with a further increase in the adsorbent dosage to 10 mg, no significant improvement in CFZ adsorption efficiency (98.1%) was achieved. Furthermore, considering the adsorption capacity values, 5 mg of adsorbent was selected as the optimal dosage; because excessively increasing the adsorbent dosage is not economically viable and contributes to a longer duration of contact between the adsorbent and the adsorbate^39^.

#### Effect of initial CFZ concentration

The dependence of the adsorption process on the initial CFZ concentration, using the MC-0.9 adsorbent, was investigated using various concentrations of the antibiotic pollutant (50 to 80 mg/L) in a batch system. Figure 4e illustrates that the adsorption efficiency of CFZ exhibited an increase with contact time, reaching a steady state after 60 min. Figure 4f also indicates a decrease in the adsorption efficiency and an increase in the adsorption capacity of the adsorbent with increasing initial CFZ concentration. This can be attributed to the greater number of available active adsorption sites for the adsorbate molecules at lower concentrations, in which case the efficiency of the adsorption process will also be higher^37,40^. However, as the initial concentration increases, the available active sites become saturated more quickly, leading to a lower percentage of removal. Despite the lower efficiency, the total amount of adsorbate adsorbed per unit mass of adsorbent increases, because more adsorbate is available in the solution^37,41^. Consequently, a concentration of 50 mg/L with an efficiency of 96.3% and an equilibrium adsorption capacity of 481.5 mg/g was selected as the optimal value.

#### Effect of solution pH

**Fig. 5:**
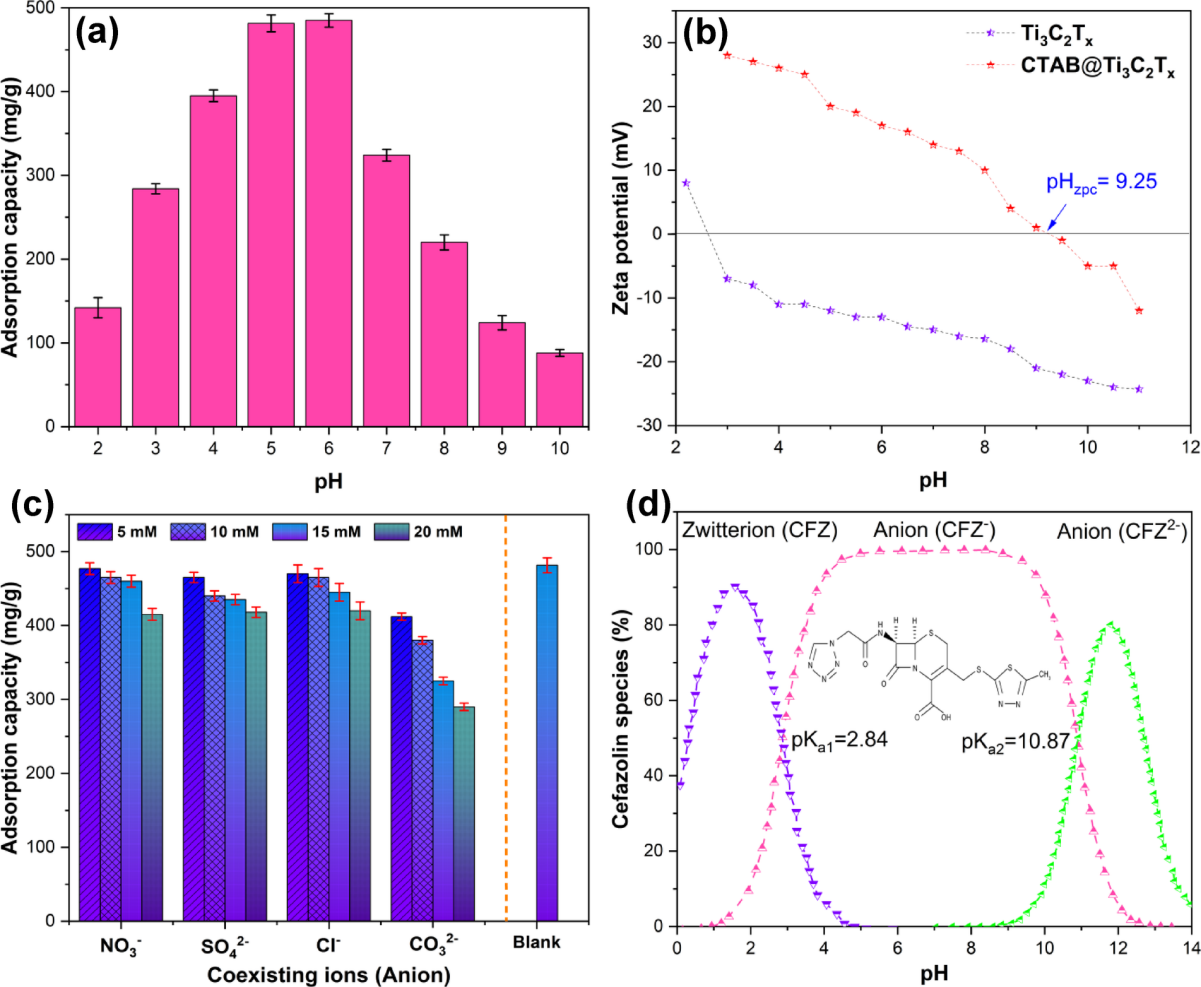
(**a**) The distribution of the zeta potential of the MXene and CTAB@MXene in aqueous solution, (**b**) CFZ species at different pH values, (**c**) effect of solution pH on the adsorption capacity of CTAB@MXene, (d) effect of coexisting anions on CFZ adsorption by CTAB@MXene (Experimental conditions: [CFZ]_0_=50 mg/L, adsorbent dosage = 5 mg, pH = 5, V = 50 mL, and contact time = 60 min).

Based on the obtained data, the point of zero charge (pH_pzc_) determination was shown in Fig. 5a. This figure illustrates the zeta potential of the pure and modified MXene adsorbent with the CTAB. As is evident, the surface of the MXene sheets exhibited a negative charge within the pH range of 2.5–11, whereas after surface modification with the cationic surfactant, the zeta potential increased within this pH range, and at pH values lower than 9.25, the surface charge of the CTAB@MXene adsorbent is positive. This may be attributed to the displacement of the naturally occurring counterions on the MXene surface by the positively charged head group of the CTAB surfactant. The observed shift in zeta potential and the change in surface charge from negative to positive at lower pH after CTAB modification indicates the successful adsorption of the cationic surfactant onto the MXene. This surface modification can significantly impact the adsorption properties of the MXene for various applications, particularly in the removal of pollutants from aqueous solutions^42^.

The impact of solution pH in the adsorption of CFZ onto the MC-0.9 adsorbent was studied within the pH range of 2 to 10. Figure 5b shows that the adsorption capacity increases by increasing pH from 3 to 6 and decreases at pH values higher than 6. The speciation of CFZ in aqueous solution is pH-dependent due to the presence of a carboxyl group (pK_a1_ = 2.84) and a secondary amid group (pK_a2_ = 10.84). At pH values below 2.84, the CFZ molecule exists as a zwitterion. Between pH 2.84 and 10.84, the anionic form (CFZ^−^) predominates, while at pH values above 10.84, the dianionic form (CFZ^2−^) is the predominant species due to the deprotonation of the H⁺ from both the amide and carboxyl groups (Fig. 5c)^11^. According to the pH_pzc_ determination in Fig. 5a, when pH < 9.25, the adsorbent surface has a positive charge, which leads to a suitable adsorption rate within the solution pH range of 2 to 6. This is due to the increased electrostatic interaction in this range. Conversely, when pH > 9.25, both the adsorbent surface and the CFZ species have a negative charge, and therefore, due to electrostatic repulsion, both the removal percentage and the adsorption capacity decrease. Consequently, based on the obtained results, the optimal pH was selected as the natural pH of the CFZ solution (~ 5) to enable further studies and investigations without the need for pH adjustment and the associated cost reduction.

#### Effect of co-existence of different anions

According to the existence of various types of salts in wastewater that affect the adsorption process, it is important to study the effect of interfering with ions on pollutant removal from aqueous solutions. Therefore, the competitive adsorption between CFZ and interfering anions, including Cl^−^, NO_3_^−^, SO_4_^2−^, and CO_3_^2−^ was investigated. The results obtained in Fig. 5d show that in solutions containing interfering compounds, the adsorption capacity of CFZ decreased with increasing concentrations of chloride, sulfate, nitrate, and carbonate anions. The order of their effect from highest to lowest is followed as: CO_3_^2−^ > Cl^−^ > SO_4_^2−^ > NO_3_^−^. The presence of the carbonate anion had a significant effect, causing the greatest reduction in the CFZ adsorption capacity, which is consistent with the other experiments^43,44^. The anions form stable complexes with the adsorbent surface, which prevents CFZ adsorption. Furthermore, the smaller size of the anions compared to the antibiotic molecule can be considered effective, which results in competition between them for adsorption onto the active adsorption sites at the optimal solution pH^45^.

### Kinetic study

The kinetic modeling was performed to find out more information about the adsorption mechanism of CFZ over MC-0.9. In addition, one important factor for designing large-scale practical batch systems is the determination of the adsorption rate, which can be explained by kinetic modeling. Non-linear regression was employed to determine the best kinetic model to fit the experimental data. Pseudo-first-order, pseudo-second-order, Elovich, and intra-particle diffusion models are widely used for estimating adsorption kinetics. The adequacy of each kinetic model in explaining the equilibrium data from the experiments was performed using four different error functions. The error equations that were implemented are listed in the supplementary file^46^.

The Pseudo-first order (PFO) model assumes that the adsorption rate is directly related to the differences between adsorption capacity at equilibrium and time t. In addition, this model supposes that the rate-determining step in the adsorption is liquid film diffusion^47^. The non-linear form of the PFO model is expressed as follows:

3 $$\:{q}_{t}={q}_{e}(1-{e}^{-{k}_{1}t})$$

The Pseudo-second order (PSO) model, one of the most employed models for describing TC adsorption, suggests that the adsorption rate is related to the square power of pollutant concentration. The non-linear form of the PSO model is expressed as follows:

4 $$\:{q}_{t}=\frac{{K}_{2}{q}_{e}^{2}t}{1+{K}_{2}{q}_{e}t}\:\:$$

The Elovich (EL) model assumes that the active sites are distributed heterogeneously over the adsorbent surface and the adsorption proceeds via chemical reactions^48^. This model is written as follows:

5 $$\:{q}_{t}=\frac{1}{\beta\:}Ln\left(\alpha\:\beta\:t+1\right)$$

Valuable information can be obtained by the intra-particle diffusion (IPD) model about the diffusion mechanism, revealing that internal or external diffusion is involved in the adsorption. This model supposes that three successive steps are included in the adsorption process: (I) pollutant diffusion from liquid bulk to the adsorbent surface, namely film diffusion, (II) pollutant diffusion to the adsorbent pores, namely intra-particle diffusion, and (III) pollutant adsorption over the active sites. This model suggests that the rate-determining step is intra-particle diffusion and film diffusion can be neglected^49^. The formula of the IPD model is described as:

6 $$\:{q}_{t}={K}_{p}{t}^{0.5}+I$$

The outcomes of the non-linear kinetic model fitting are depicted in Fig. S2, while the kinetic parameters and the corresponding error values are provided in Table 1. As seen, the PSO model demonstrated the best agreement with the experimental data, compared to the other models. In addition, the q_e_ calculated by PSO was very similar to the values obtained experimentally in all adsorbent dosages. Generally, different factors including external film diffusion and particle diffusion are included in the PSO model. So, this model is more suitable for describing the adsorption experimental data in aquatic systems. Moreover, the values for I (the intercept of the IPD model) were not obtained zero, revealing that different mechanisms are effective on the CFZ adsorption, besides intra-particle diffusion.

**Table 1:** Kinetic parameters for CFZ adsorption on the prepared adsorbent.

Kinetic model	Parameter	Initial concentration (mg/L)			
		50	60	70	80
Experimental	(q_e) Exp_. (mg/g)	482.5	516.1	539.4	552.8
PFO	(q_e_)_Cal_. (mg/g)	577.5	625.1	637.9	632.9
	k_1_ (min^− 1^)	0.057	0.059	0.063	0.080
	R^2^	0.981	0.972	0.974	0.947
	RMSE	16.54	21.12	19.45	23.48
	AARE	1.592	2.144	2.014	3.696
	STD	0.020	0.032	0.028	0.048
PSO	(q_e_)_Cal_. (mg/g)	486.2	524.1	539.3	547.7
	K_2_ (g/mg/min)	0.0010	0.0011	0.0013	0.0015
	R^2^	0.996	0.992	0.994	0.989
	RMSE	16.42	18.51	17.94	19.35
	AARE	1.844	2.089	1.907	2.146
	STD	0.021	0.24	0.023	0.026
IPD	k_p_ (mg/g/min^0.5^)	47.13	50.65	51.65	52.19
	I (mg/g)	75.93	77.52	90.84	126.9
	R^2^	0.828	0.816	0.801	0.718
	RMSE	19.41	21.79	28.46	33.73
	AARE	2.277	2.801	2.887	3.199
	STD	0.057	0.049	0.051	0.069
Elovich	α (g/mg/min)	70.63	69.71	84.72	141.8
	β (mg/g/min)	0.0079	0.0077	0.0076	0.0071
	R^2^	0.945	0.933	0.932	0.893
	RMSE	22.47	24.43	25.11	34.59
	AARE	1.915	2.679	2.877	3.771
	STD	0.024	0.032	0.035	0.052

### Isotherm study

Accurate assessment of adsorption processes relies on equilibrium data, as these data fully characterize the separation between solid and liquid phases at equilibrium. This characterization is crucial for elucidating the adsorption of adsorbed molecules from the solution to the sorbent surface. The CFZ adsorption data onto MC-0.9, which are each described by Eqs. (7)-(10), were fitted to four famous non-linear mathematical models: Langmuir, Freundlich, Temkin, and Dubinin-Radushkevich. The Langmuir model assumes that monolayer adsorption occurs over the active sites distributed homogenously on the adsorbent surface. Also, based on this model, each molecule occupies only one site without any interaction between the adsorbate molecules. Moreover, the active sites bear uniform energies^50^. The non-linear form of the Langmuir model is expressed as follows:

7 $$\:{q}_{e}=\frac{{q}_{max}{K}_{L}{C}_{e}}{1+{K}_{L}{C}_{e}}$$

The Freundlich model, a common isotherm employed in different research, assumes that multilayer adsorption occurs over the active sites distributed heterogeneously on the adsorbent surface. In addition, it is assumed that the adsorption enthalpy declines logarithmically as the amounts of occupied sites increase^51^. The non-linear form of the Freundlich model is expressed as follows:

8 $$\:{q}_{e}={K}_{F}{{C}_{e}}^{\frac{1}{n}}$$

If n is obtained in the range of 1 to 10, it means that the conditions are satisfactory for adsorption.

Based on the Tempkin model, the heat of adsorption drops as the surface coverage increases^52^. The non-linear formula of the Tempkin model is written as follows:

9 $$\:{q}_{e}=\frac{RT}{{b}_{T}}Ln\left({K}_{T}{C}_{e}\right)$$

The Dubinin-Radushkevich (DR) model describes that the adsorption occurs over the heterogenous surface in which the adsorption energies are distributed in Gaussian type. In addition, this model assumes that the adsorption curve is highly influenced by the textural properties of the adsorbent^53^. The non-linear formula of the DR model is expressed as follows:

10 $$\:{q}_{e}={q}_{d}{e}^{-{\epsilon\:}^{2}{K}_{DR}}$$

11 $$\:\epsilon\:=RTLn\left(1+\frac{1}{{C}_{e}}\right)$$

12 $$\:E=\frac{1}{\sqrt{2{K}_{DR}}}$$

The value of E (kJ/mole) indicates the physical or chemical properties of the adsorption. 8 < E < 16 implies the chemisorption, and a value lower than 8 kJ/mol is indicative of the physisorption.

Figure S3 depicts the nonlinear isotherms for the isotherm models. The parameters of different isotherm models and their error values are provided in Table 2. The results indicated that the Langmuir model was more successful in predicting experimental data. Therefore, adsorption occurred monolayer over the homogenous surface of the MC-0.9. In addition, the value of 5.61 was obtained for n, which indicates that the CFZ adsorption was favorable. The DR model analysis revealed an E value of 0.69 kJ/mol, confirming that the dominant mechanism for CFZ adsorption onto the cationic surfactant-modified MXene adsorbent is physisorption (E < 8 kJ/mol).

**Table 2:** Adsorption isotherm parameters for CFZ removal at different initial CFZ concentrations (Experimental conditions: adsorbent dosage = 0.1 g/l, pH = 5, V = 50 mL, [CFZ]_0_=50–80 mg/L).

Isotherm model	Parameter	Value
Langmuir	Q_0_ (mg/g)	575.1
	K_L_ (L/mg)	1.12
	R^2^	0.997
	RMSE	15.34
	AARE	1.457
	STD	0.019
Freundlich	K_F_ (L/g)	331.2
	n	5.61
	R^2^	0.864
	RMSE	21.54
	AARE	2.692
	STD	0.089
Tempkin	K_T_ (L/g)	0.174
	b_T_	42.18
	R^2^	0.963
	RMSE	19.11
	AARE	2.541
	STD	0.071
Dubinin–Radushkevich	K_DR_	1.056
	E (kJ/mol)	0.69
	q_d_ (mg/g)	534.2
	R^2^	0.995
	RMSE	17.54
	AARE	2.192
	STD	0.033

### Adsorption mechanism and comparison with other adsorbents

Total Organic Carbon (TOC) analysis provides a more holistic view of organic pollutant removal, as it measures the total organic carbon content in the solution. This is crucial for assessing the overall effectiveness of the adsorption process in reducing the organic load of wastewater^54^. As shown in Fig. S4, the TOC removal rates closely correlated with the high CFZ removal efficiencies observed via UV-Vis. The close correlation between the two analyses indicated that the CFZ molecules were remaining intact during the adsorption process with minimal degradation or transformation of CFZ into other organic byproducts. The consistency of these results strongly suggests that the primary mechanism of removal is adsorption, where the pollutant molecules are being transferred from the solution to the solid adsorbent, and that the organic load of the water is being reduced.

**Fig. 6:**
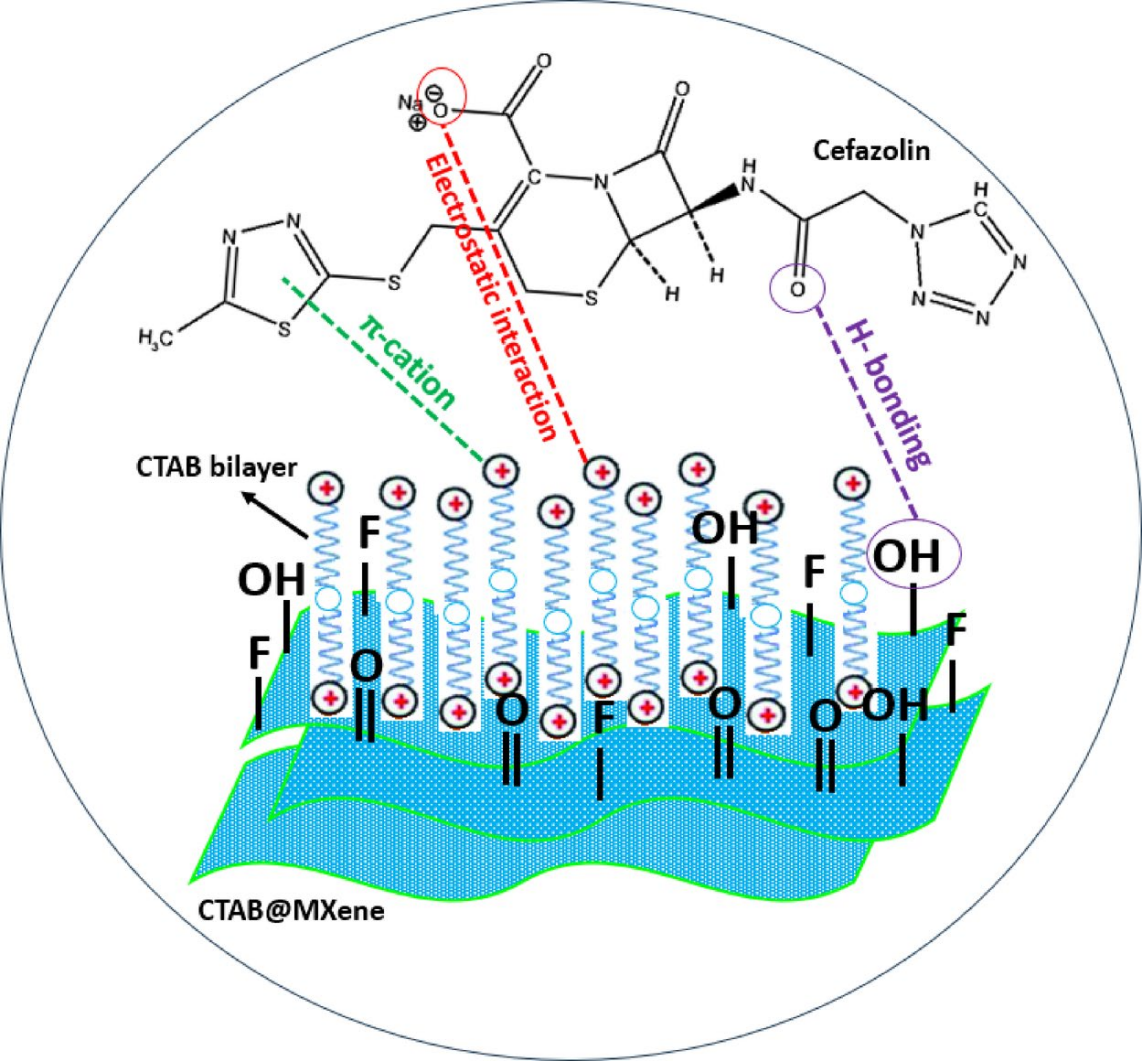
Schematic illustration of the CFZ adsorption mechanism by CTAB@MXene.

The primary mechanism for the adsorption of CFZ onto CTAB@MXene adsorbent is attributed to electrostatic interactions. The negatively charged Ti_3_C_2_T_x_ nanosheets interact with the cationic surfactant CTAB, which enhances the adsorption capacity by creating an electrostatic attraction between the CFZ and the modified MXene surface. As previously mentioned, CFZ exhibits pH-dependent speciation. In the pH range of 2.84 to 10.84, CFZ exists primarily in its anionic form (CFZ^−^), possessing a net negative charge. This negative charge arises from the deprotonation of the carboxyl group. On the other hand, the presence of the cationic surfactant CTAB, which introduces positively charged quaternary ammonium groups, leads to the favorable electrostatic attraction between CFZ^−^ and the positively charged CTAB-modified MXene surface. In addition to electrostatic interactions, hydrogen bonding plays a significant role in the adsorption process. The primary functional groups in CFZ include a β-lactam ring, a thiazole ring, and an acetamido group^55,56^. Hydrogen bonding occurs between the functional groups present on the CFZ and the surface of the CTAB@MXene, facilitating the attachment of antibiotic molecules. Moreover, the π-cation interaction occurs when the positively charged head groups of CTAB interact with the negatively polarized π-electron cloud of the CFZ’s thiazole ring. This further confirms the enhanced binding affinity between the antibiotic and the adsorbent. The modification of Ti_3_C_2_T_x_ MXene with CTAB increases the distance between the MXene nanosheets (see XRD analysis), which exposes more active sites for adsorption. Moreover, the pore distribution of CTAB@MXene was broadened (See BET analysis). This structural change enhances the overall effectiveness of the adsorbent in capturing the CFZ molecules from the aqueous solution. Schematic illustration of the CFZ adsorption mechanism is presented in Fig. 6.

The maximum adsorption capacity of 481.5 mg/g, as determined by the Langmuir model, was compared with reported values from similar systems in Table 3. While the current system demonstrates adsorption capabilities, the comparison reveals that further optimization may be necessary to achieve comparable performance. As can be seen, our developed system containing CTAB@MXene exhibits substantially outstanding adsorption capacity. Therefore, it was deduced that the prepared adsorbent represented appropriate potential as an efficient adsorbent for the removal of CFZ and other pollutants.

**Table 3:** Comparative studies of CFZ removal between different adsorbents.

Sample	Adsorbent dosage (g/L)	Initial concentration of CFZ (mg/L)	pH	Contact time (min)	Temperature (°C)	Removal (%)	Q_0_ (mg/g)	References
Chitosan/EC	0.5	60	7.5	23	25	100	1250	^25^
Fe/amine modified Chitosan	0.6	45	6	2880	25	-	341	^13^
Spectrogel^®^ organoclay	8.96	60	5	4320	25	80	398.6	^11^
Mt/nZVI/GO	0.4	5	7	10	25	86	20.36	^57^
Electrosynthesized Mg(OH)_2_	–	2.5	7	60	30	85.2	12.48	^58^
Fe_3_O_4_/AC	0.3	20	6	150	25	96	123.46	^39^
CdS-MWCNT	0.45	25	8	15	20	–	34.5	^59^
PA Resin-PEG	3	100	5	60	25	98	–	^60^
CTAB@MXene	0.1	50	5	60	25	96.3	575.1	This study

### Regeneration study

**Fig. 7:**
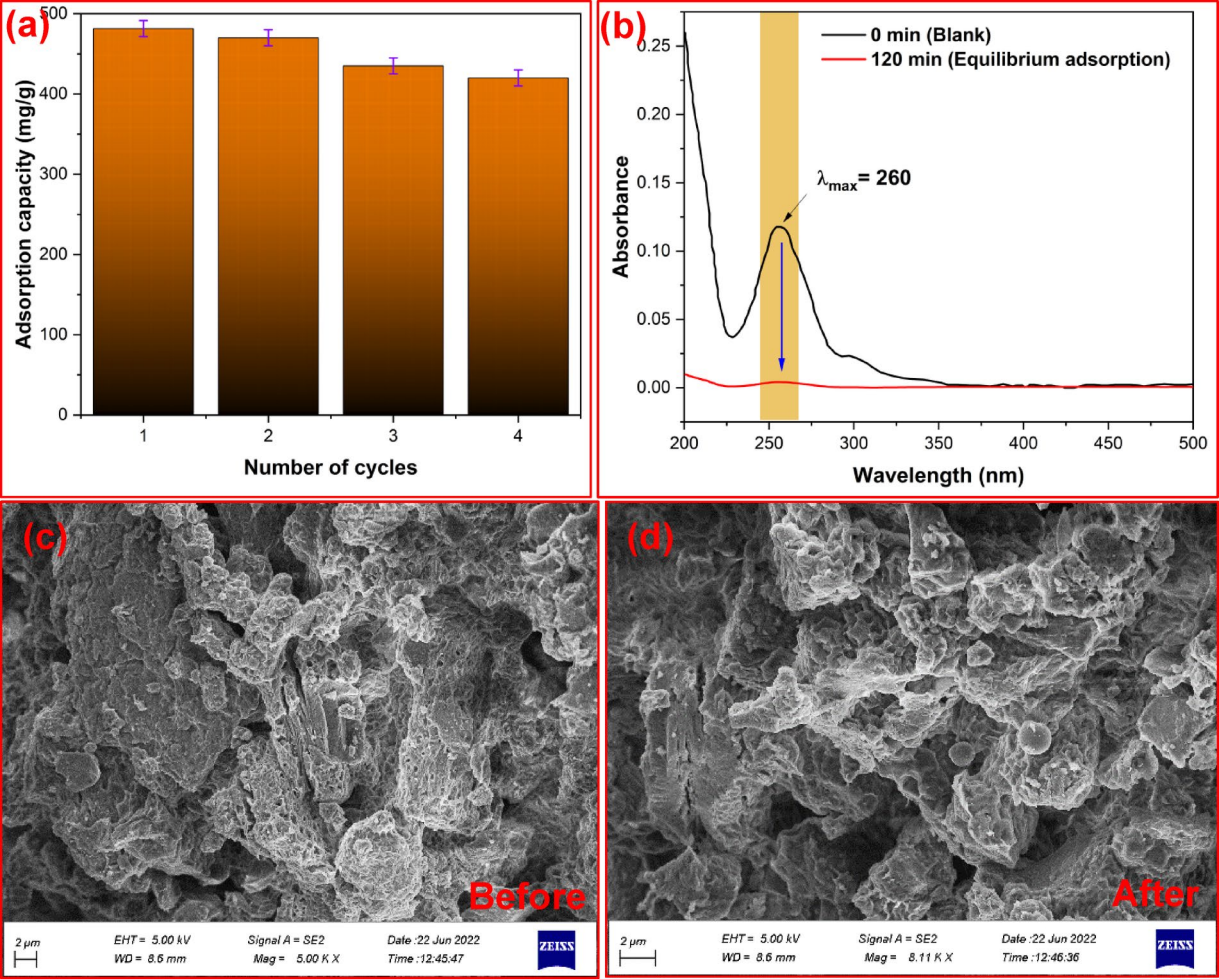
(**a**) Adsorption mechanisms during CFZ removal using CTAB@MXene, (**b**) recyclability of the adsorbent, and (**c**,**d**) SEM images of MC-0.9 adsorbent before and after regeneration.

Recyclability and reusability of an adsorbent are crucial parameters for its selection and industrial application. Therefore, the stability of the MC-0.9 adsorbent was evaluated through several consecutive adsorption cycles. After each adsorption cycle under the optimized conditions (adsorbent dosage 5 mg, solution pH 5, solution volume 50 mL, initial antibiotic concentration 50 mg/L, and contact time 60 min), the adsorbent was separated from the wastewater solution using centrifugation. Then it was regenerated by washing several times with deionized water and ethanol, followed by drying at 75 °C for 12 h. The dried adsorbent was then utilized in the next batch adsorption cycle. The results obtained in Fig. 7a showed that the equilibrium adsorption capacity of CFZ decreased from 481.5 to 421.3 mg/g after four consecutive uses, demonstrating the reusability and recyclability of the adsorbent. The slight decrease in adsorption capacity and adsorbent performance can be attributed to the detachment of the cationic surfactant from the MXene surface due to repeated washing. Figure 7b shows the CFZ absorption spectra of the fourth cycle. To further evaluate the adsorbent stability, FESEM analysis was performed on the adsorbent before and after usage and the results are compared in Fig. 7c and d. As seen, the morphology of the adsorbent was preserved after regeneration in the adsorption process and remained largely unchanged.

### Toxicity assessment

To evaluate the acute toxicity of the treated effluent, we employed *Daphnia magna*, a crustacean zooplankton widely recognized as a sensitive bioindicator in both freshwater and marine environments. Commercially sourced *D. magna* eggs were hatched and cultured under controlled laboratory conditions to ensure a standardized population for testing. Acute toxicity assays were conducted in triplicate within sterile plastic containers, each containing 15 healthy *D. magna* individuals and 5 mL of the treated effluent sample. A series of dilutions were prepared, representing 0%, 10%, 25%, 50%, 75%, and 100% volume percentages of the treated effluent, to determine the concentration-dependent toxicity. The exposure period extended over 6, 12, 24, 48, and 72 h, allowing for a comprehensive assessment of time-dependent toxicity. Following each exposure period, the number of deceased *D. magna* individuals was meticulously recorded, adhering to established toxicological protocols^61^. The resulting mortality data, presented in Table 4, revealed a relatively high tolerance of *D. magna* to the treated effluent. Significant mortality was only observed after 48 h of exposure to undiluted (100% v/v) effluent, and after 72 h of exposure to 75% and 100% v/v effluent. This suggests that while the treated effluent exhibits some acute toxicity at high concentrations and prolonged exposure, it demonstrates a reduced overall toxicity profile.

**Table 4:** Daphnia toxicity assessment of adsorbed-CFZ solutions.

Dose (V/V_0_) %	Total Daphnia	Number of Daphnia dead during the test				
		6 h	12 h	24 h	48 h	72 h
0	15	0	0	0	0	0
10	15	0	0	0	0	0
25	15	0	0	0	0	0
50	15	0	0	0	0	2
75	15	0	0	0	0	3
100	15	0	0	0	2	4

According to the Ecotoxicological assessment of Ti_3_C_2_T_*x*_ (MXene) by G. K. Nasrallah et al.^62^, it can be classified as within the practically nontoxic group according to the acute toxicity rating scale by the fish and wildlife service (FWS); thus, the use and discharge of Ti_3_C_2_T_*x*_ MXene in the aquatic ecosystem at concentrations below 100 µg mL^− 1^ is safe. Here, in this study the optimum amount of the adsorbent dosage was 5 mg/ 50 mL (100 µg mL^− 1^). So, after separating the adsorbent from the liquid media, the remaining amount of adsorbent is certainly very lower than 100 µg mL^− 1^. Moreover, the TOC analysis was performed to assess the CTAB leaching after different adsorption cycles (Table S1). The low increase of TOC concentration indicated the slight detachment of the cationic surfactant from the MXene surface.

## Conclusions

In this study, the MXene phase was initially synthesized by etching the middle layer (aluminum) of the MAX phase, and then its surface was modified with the cationic surfactant CTAB. Different techniques such as FESEM, EDS, FTIR, XRD, BET, and zeta potential methods were employed to characterize the synthesized samples. The results showed that 0.9 mM was the optimal CTAB concentration for surface modification of the MXene phase, and the removal efficiency of CFZ was enhanced by using the cationic surfactant from 22% (pure MXene) to 96.3% (MC-0.9). Based on the dissociation constants of CFZ, its anionic species, and the pH_pzc_ of the CTAB@MXene adsorbent, the optimal pH for the removal of this pharmaceutical pollutant was found to be 5. Accordingly, under the optimum operating conditions (adsorbent dosage 0.1 g/L, solution pH 5, initial CFZ concentration 50 mg/L, and contact time 60 min), the removal efficiency of 96.3%, and the highest adsorption capacity of 481.5 mg/g were achieved. The coexistence of inorganic anions exhibited a decrease in adsorption capacity with the following order of influence: CO_3_^2^^−^ > Cl^−^ > SO_4_^2^^−^ > NO_3_^−^. Among the effective mechanisms in the absorption of CFZ, hydrogen bonding, electrostatic and π-cation interactions had the most effect on the adsorbent performance. The adsorption data of CFZ in a batch system were well-fitted with the pseudo-second-order kinetic and the Langmuir isotherm models. The use of the MC-0.9 adsorbent in four consecutive adsorption cycles demonstrated its suitable stability. Finally, the toxicity of effluent was assessed, and the results indicated that the effluent does not create any environmental concerns.

## Electronic supplementary material

Below is the link to the electronic supplementary material.

Supplementary Material 1

## Data Availability

The datasets used and/or analyzed during the current study are available from the corresponding author on reasonable request.
