# Supplementary material for: Surface modification of MXene using cationic CTAB surfactant for adsorptive elimination of cefazolin antibiotic from water
Source: Sci Rep. 2025 May 12;15:16416. doi: 10.1038/s41598-025-01435-y (PMC12069570; doi:10.1038/s41598-025-01435-y)
Supplement: Supplementary file 1 — Supplementary Material 1 [file 41598_2025_1435_MOESM1_ESM.docx]

**Supporting Information**

**Surface modification of MXene using cationic CTAB surfactant for adsorptive elimination of cefazolin antibiotic from water**

Jafar Abdi ^a, b, *^, Golshan Mazloom ^c^, Yeojoon Yoon ^d^

**^a^** Faculty of Chemical and Materials Engineering, Shahrood University of Technology, 3619995161 Shahrood, Iran

^b^ Center for International Scientific Studies and Collaborations, Tehran, Iran

^c^ Department of Chemical Engineering, Faculty of Engineering, University of Mazandaran, 47416-13534 Babolsar, Iran

^d^ Department of Environmental and Energy Engineering, Yonsei University, Wonju, Republic of Korea

Corresponding authors (*):

[Jafar.abdi@shahroodut.ac.ir](mailto:Jafar.abdi@shahroodut.ac.ir) (J. Abdi)

**Evaluation of the kinetic and isotherm models quality**

The quality and reliability of the kinetic and isotherm models were assessed using different statistical techniques described as follows:

1. The average absolute relative deviation of the model results from the experimental values was calculated by the average absolute relative error (AARE):

$AARE\left( \% \right)=\frac{100}{n}\sum_{i=1}^{n} \left| \frac{X{(i)}_{model}-X{(i)}_{exp}}{X{(i)}_{exp}} \right|$

2. The root mean square error (RMSE), which indicates the error dispersion, is calculated by:

$RMSE=\left( \frac{\sum_{i=1}^{n} (X\left( i \right)_{model}-X{(i)}_{exp})^{2}}{n} \right)^{\frac{1}{2}}$

3. The dispersion of data is investigated by the standard deviation of errors (STD), which can be calculated using:

$STD=\frac{1}{n}\sum_{i=1}^{n} {\left( X\left( i \right)_{model}-\bar{X(i)}_{model} \right)^{2})}^{\frac{1}{2}}$

4. The coefficient of determination $\left( R^{2} \right)$ which assigns the accuracy of the predictions. The $R^{2}$ value close to 1 determines that the estimation of experimental data is more accurate.

$R^{2}=1-\frac{\sum_{i=1}^{n} \left( X{(i)}_{model}-X{(i)}_{exp} \right)^{2}}{\sum_{i=1}^{n} \left( X{(i)}_{model}-\bar{X(i)}_{exp} \right)^{2}}$


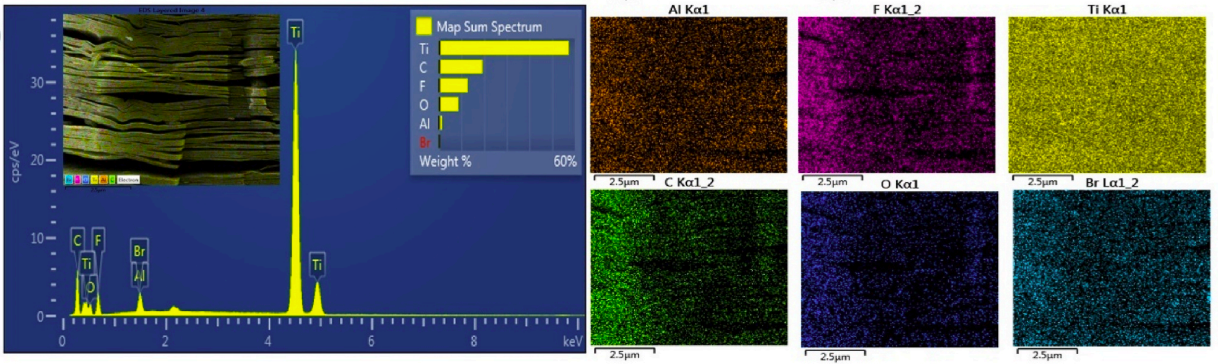


**Fig. S1.** EDS spectrum and elemental mapping of CTAB@MXene.


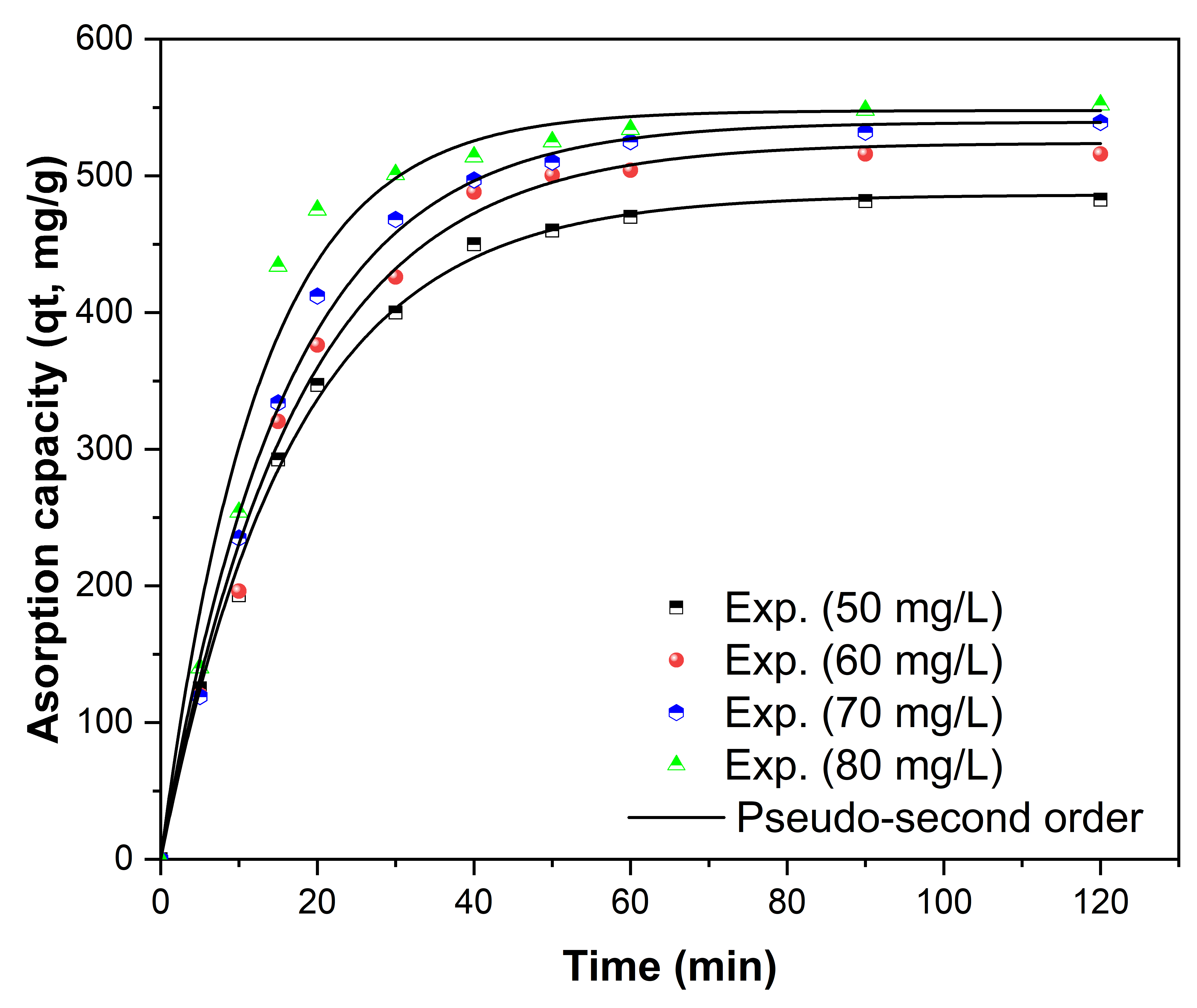

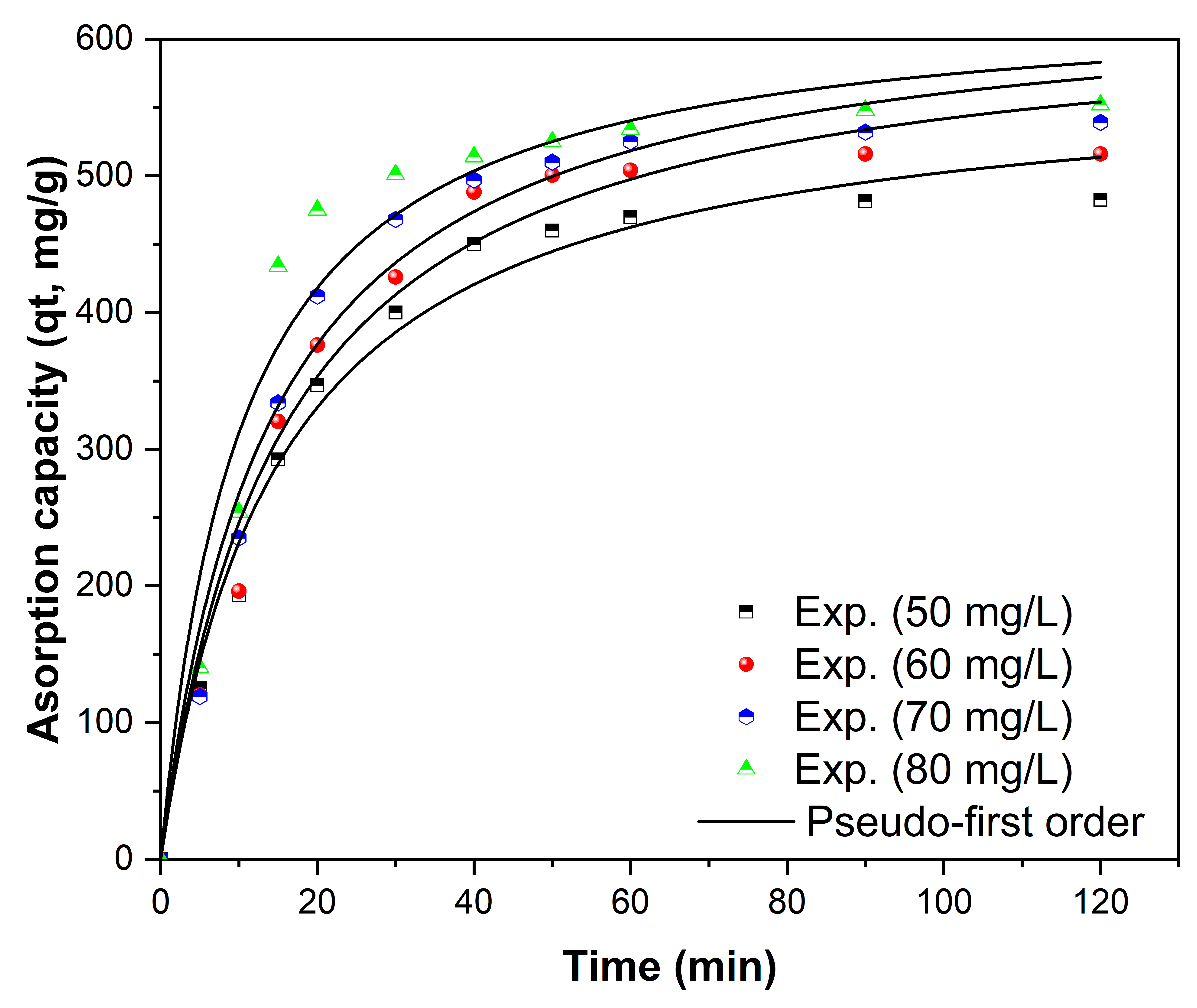


**(b)**

**(a)**

| **Fig. S2**. Non-linear kinetic plots for CFZ adsorption onto CTAB@MXene. |
| --- |


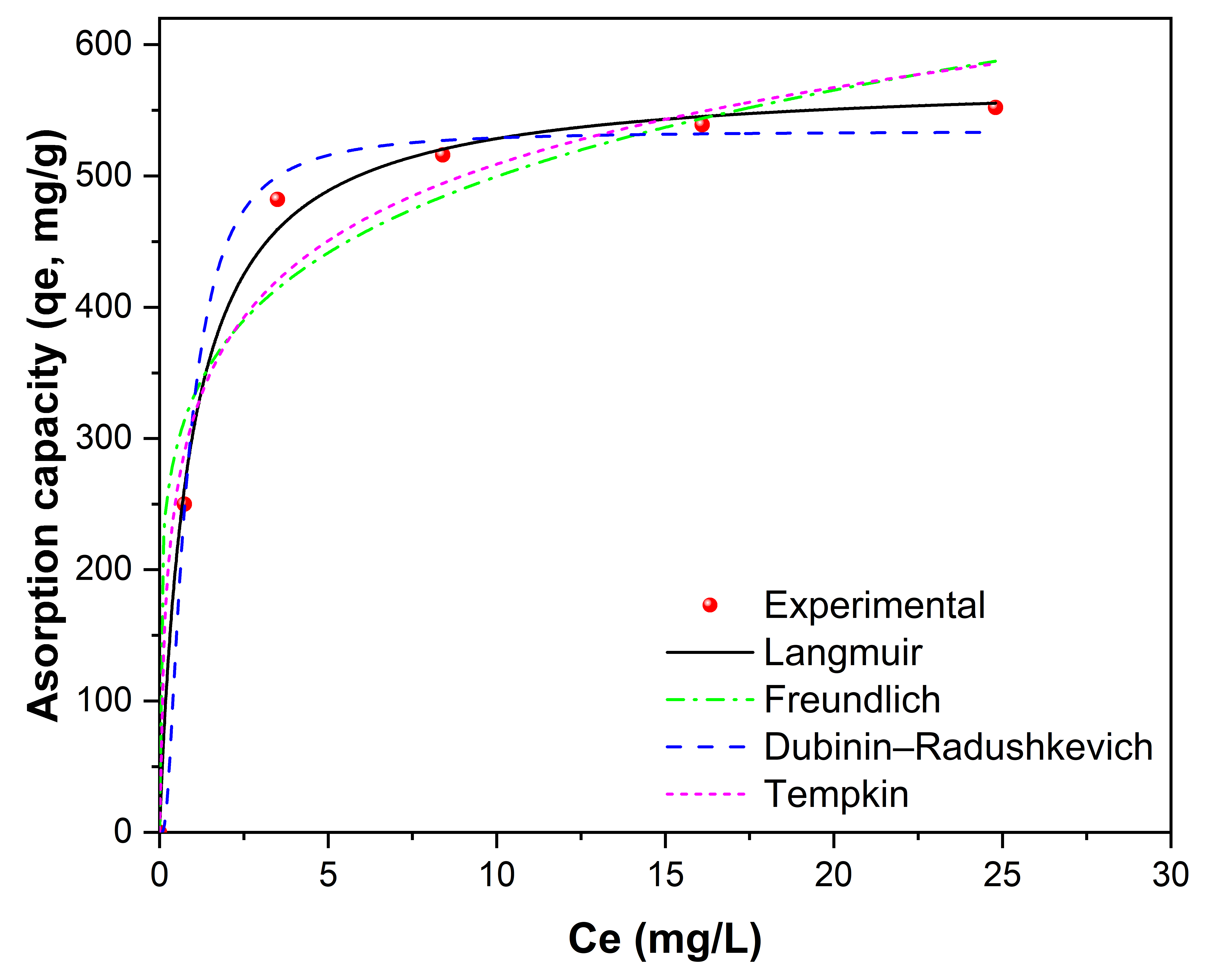


**Fig. S3.** Non-linear adsorption isotherm plots for CFZ adsorption onto CTAB@MXene.


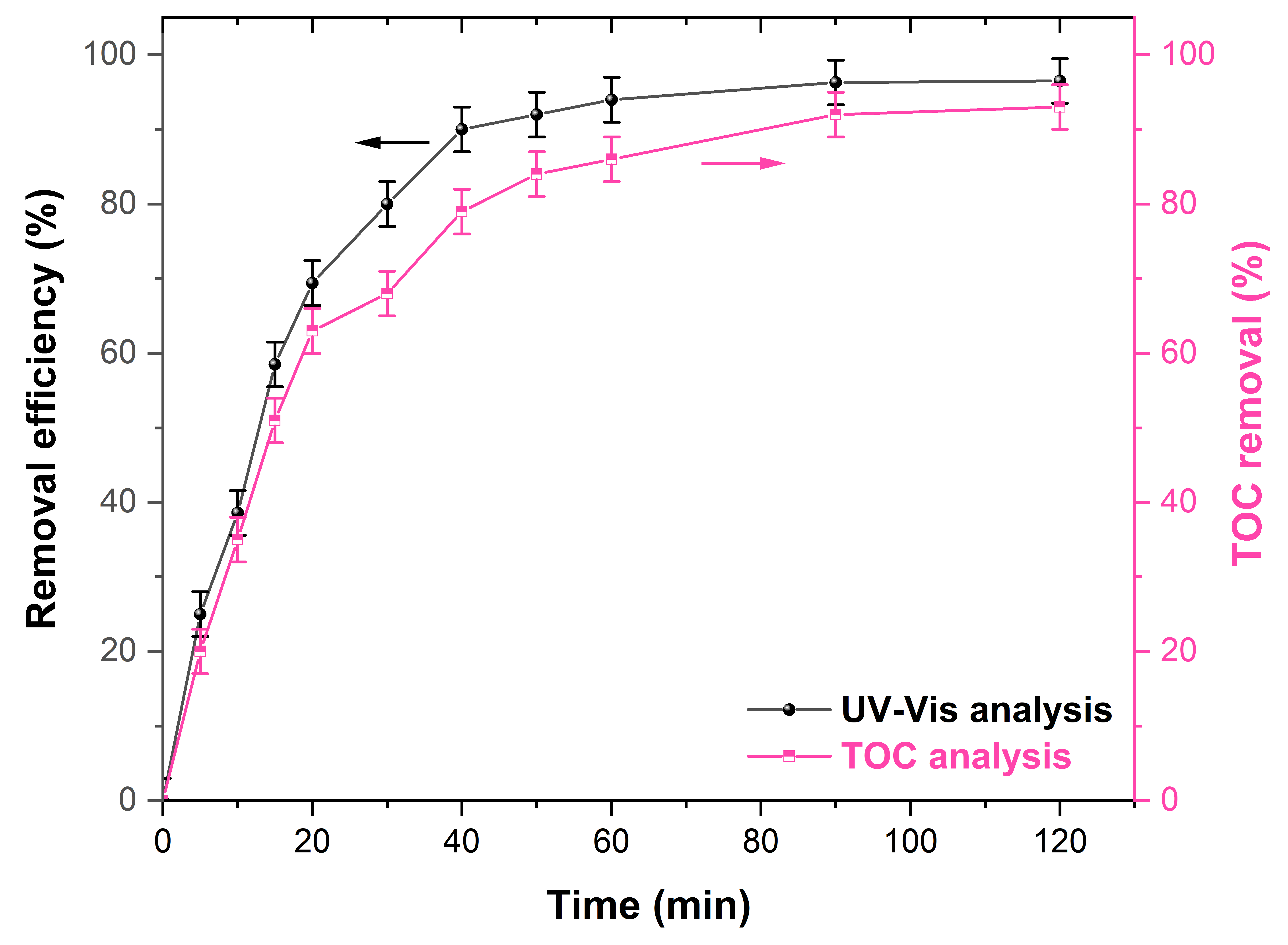


**Fig. S4.** TOC removal rates and adsorption efficiencies observed via UV-Vis for CFZ removal.

**Table S1.** The results of TOC analysis from the remaining CFZ solution after different adsorption cycles under the optimized conditions.

| Sample | [TOC]_0_ (mg/L) |
| --- | --- |
| Pure water | 5.21 |
| First cycle | 11.7 |
| Second cycle | 13.8 |
| Third cycle | 14.6 |
| Forth cycle | 15.2 |
